# ﻿Mountainous millipedes in Vietnam. V. The millipede genus *Kronopolites* Attems, 1914 (Diplopoda, Polydesmida, Paradoxosomatidae), with descriptions of two new species

**DOI:** 10.3897/zookeys.1249.155280

**Published:** 2025-08-25

**Authors:** Anh D. Nguyen, Tam T. T. Vu, Hong Luong T. Phung, Duc-Luong Tran, Hung-Anh Le

**Affiliations:** 1 Institute of Biology, Vietnam Academy of Science and Technology, 18, Hoangquocviet Rd., Caugiay District, Hanoi, Vietnam Graduate University of Science and Technology Hanoi Vietnam; 2 Graduate University of Science and Technology, Vietnam Academy of Science and Technology, 18, Hoangquocviet Rd., Caugiay District, Hanoi, Vietnam Vietnam Academy of Science and Technology Hanoi Vietnam

**Keywords:** Biodiversity, COI, new species, Southeast Asia, taxonomy

## Abstract

The paradoxosomatid genus *Kronopolites* Attems, 1914 has been reviewed in the context of Vietnam’s fauna. A total of six species have been documented in the country, including a new country record, *K. biagrilectus* Hoffman, 1963, and two new species, *K. contrastus***sp. nov.** and *K. serratus***sp. nov.** The genetic distances and the phylogenetic relationship between Vietnamese *Kronopolites* species based on a fragment of the COI gene were also provided.

## ﻿Introduction

Situated in mainland Southeast Asia, Vietnam is recognized for its rich biodiversity, including a diverse millipede fauna. To date, approximately 280 millipede species belonging to 97 genera, 31 families, and 14 orders have been recorded from Vietnam (Nguyen et al. 2025). Among these, the family Paradoxosomatidae is the most dominant, comprising 116 species in 29 genera. Globally, it is also the most species-rich millipede family, with more than 1,000 described species (Nguyen and Sierwald 2013).

Within the family Paradoxosomatidae, *Kronopolites* Attems, 1914 is a small genus with 14 species distributed in temperate Asia ranging from the Himalayas of Kashmir, India in the west to Taiwan in the east (Table 1). Attems (1914) established the genus to accommodate the species *Strongylosoma swinhoei* Pocock, 1895. However, Xiong et al. (2025) recently amended its type species, formally transferring *Strongylosoma swinhoei* to the genus *Nedyopus* Attems, 1914, while designating *Kronopolites svenhedini* (Verhoeff, 1934) as the type species of the genus *Kronopolites*.

**Table 1.** All known *Kronopolites* species (Golovatch 2015, 2020; Likhitrakarn et al. 2015; Golovatch and Semenyuk 2021; Xiong et al. 2025).

No	Species	Location
1	*Kronopolites acuminatus* Attems, 1937	Ha Giang Prov., northern Vietnam
2	*Kronopolites biagrilectus* Hoffman, 1963	Widely distributed in southern China
3	*Kronopolites coriaceus* Golovatch, 2015	Kaski District (2000m), Nepal
4	*Kronopolites davidiani* Golovatch, 2014	Yunnan Prov. (3,365m), southern China
5	*Kronopolites formosanus* (Verhoeff, 1939)	Taipei (below 1,000m), northern Taiwan
6	*Kronopolites fuscocingulatus* Jeekel, 1982	Doi Suthep (800–900m), Doi Pha Hom Pok (1,550–1,660m), Doi Chiang Dao (1,300m), all in northern Thailand
7	*Kronopolites lunatus* Likhitrakarn, Golovatch & Panha, 2015	Xieng Khoang Prov. (1,180m) and Luang Prabang Prov. (440m), northern Laos
8	*Kronopolites montanus* Golovatch, 2009	Sapa (2,000m), northern Vietnam
9	*Kronopolites occidentalis* Golovatch, 1983	Pir Panjal Mt. (2,600m), Pari Mahal Monastery (1,500m) northern India
10	*Kronopolites ramosus* Golovatch & Semenyuk, 2021	Pu Mat NP (350m), Nghe An Prov., northcentral Vietnam
11	*Kronopolites rugosus* Golovatch, 2013	Lijiang (2,400m), Yunnan Prov., China
12	*Kronopolites semirugosus* Golovatch, 2013	Mianning (2,955m), Sichuan Prov., China
13	*Kronopolites svenhedini* (Verhoeff, 1933)	Widely distributed in mainland China
14	*Kronopolites typicus* Golovatch, 2020	Laoheshang Mt. (2,780m), Yunnan Prov., China

Despite the diverse millipede fauna of Vietnam, only three species of *Kronopolites* have been recorded in the country (Table 1). Therefore, this study aims to contribute to the knowledge of the Vietnam’s millipede diversity by reviewing the genus *Kronopolites* found in the country. A new national record and two new species have been included in the fauna of Vietnam.

## ﻿Materials and methods

Millipede specimens were collected from northern Vietnam, and preserved in ethanol 80%. Morphological characters were investigated with an Olympus SZX16 stereomicroscope. Gonopods were dissected for morphological examination and photographed. Colour images were taken at various focal planes using a camera Sony A6000 coupled with a SMZ800N Nikon stereomicroscope. UV images were taken using a Sony A6000 digital camera attached to the aforementioned SMZ800N Nikon stereomicroscope under a UV flashlight Nichia Convoy. Images were stacked using Helicon Focus v. 7.0 and assembled in Adobe Photoshop CS6. Scanning electron microscope (SEM) images were taken using the system Prisma E (ThermoFisher Scientific) in the Institute of Biology (previously known as Institute of Ecology and Biological Resources), Hanoi, Vietnam.

DNA was extracted using Qiagen DNeasy Blood and Tissue Kits. A 680–bp fragment of the mitochondrial gene, cytochrome c oxidase subunit I (COI), was amplified and sequenced using a pair of universal primers, LCO1490 and HCO2198 (Folmer et al. 1994). Polymerase chain reaction (PCR) conditions for amplification of the COI gene follow those of Nguyen et al. (2017). The successfully amplified PCR products were sent to the GenLab company (Vietnam) for purification and sequencing. COI sequences were verified using BLASTN 2.6.0+ (Zhang et al. 2000) and registered in GenBank with unique accession numbers. All verified sequences were aligned using multiple sequence alignment with the program ClustalX ver. 2 (Larkin et al. 2007). Genetic distances between samples were calculated using the Kimura 2–parameter model in MEGA ver.7.0 (Kumar et al. 2016). A maximum likelihood bootstrap analysis was conducted using the IQTREE server with 1,000 replicates at http://iqtree.cibiv.univie.ac.at/ (Trifinopoulos et al. 2016). The species *Antheromorpha festiva* (Broelemann, 1896) was selected as an outgroup.

All terminology follows Likhitrakarn et al. (2015), Golovatch (2020), and Golovatch and Semenyuk (2021). Type specimens are deposited in Myriapod Collection, Institute of Biology, Hanoi, Vietnam (**IB**).

**Abbreviations: IEBR-Myr** = Institute of Biology, Myriapod collection, Hanoi, Vietnam; **ZMUM** = Zoological Museum, University of Moscow, Russia; **NHMW** = Naturhistorisches Museum Wien, Austria; **NP** = National Park; **NR** = Natural Reserve; **Prov.** = Province.

## ﻿Results

### ﻿Taxonomic account

**Order Polydesmida Leach, 1812**

**Family Paradoxosomatidae Daday, 1889**

**Tribe Sulciferini Attems, 1898**

#### ﻿Genus Kronopolites

Taxon classificationAnimaliaPolydesmidaParadoxosomatidae

Attems, 1914

77148E6F-FFCD-55A1-8C40-426FE391D158

##### Type species.

*Kronopolites svenhedini* (Verhoeff, 1934) corrected by Xiong et al. (2025).

##### Remarks.

The genus was recently revised by Likhitrakarn et al. (2015). The genus can be recognised by the gonopod solenophore typically carrying a fork consisting of two basal processes: curved or suberect, anteriad-directed process a and posteriad-directed process b.

Most species of *Kronopolites* live in montane forest habitats usually found at elevations between ca 400 m and 2,700 m a.s.l.

#### ﻿ Kronopolites acuminatus

Taxon classificationAnimaliaPolydesmidaParadoxosomatidae

Attems, 1937

62093EBB-9A62-525C-81D2-B2CB14830E6D

Fig. 1

Kronopolites acuminatus Attems, 1937: 52; Attems 1938: 227, fig. 53; Jeekel 1968: 59; Golovatch 2009: 121, in key;
Kronopolites acuminatus acuminatus : – Hoffman 1963: 584, established new subspecific status; Golovatch 1983: 181; Enghoff et al. 2004: 38.

##### Type specimens.

NHMW.

##### Material examined.

Vietnam • **Lao Cai Province** • 1 male, 2 females; Hoang Lien National Park, on the way to Fanxipan Mt.; 22.35087°N, 103.78030°E; primary forest; 2,000 m a.s.l.; 28 September 2005; Anh D. Nguyen leg.; IEBR-Myr 115 • 1 male, 1 female; Hoang Lien National Park, Tram Ton, Tram Ton station, observation tower; 22.35347°N, 103.77541°E; secondary forest; 1,900 m a.s.l.; 23 March 2007; Anh D. Nguyen leg.; IEBR-Myr 187 • 1 male, 2 females; Bat Xat Natural Reserve; 22.61148°N, 103.64392°E; natural forest; 28 December 2017; Hung D. Nguyen leg.; IEBR-Myr 760 • 1 male, 1 female; same data as IEBR-Myr 760; IEBR-Myr 761.

**Figure 1.**
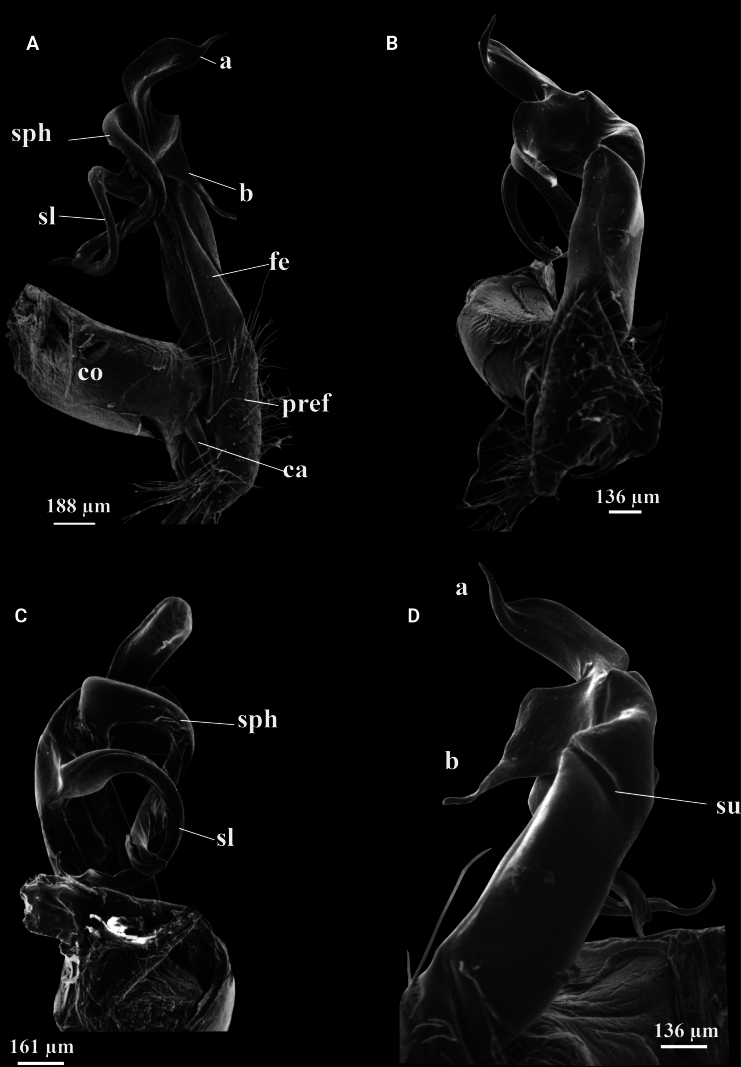
*Kronopolites acuminatus* Attems, 1937 from Lao Cai (IEBR-Myr 115). A–D. Left gonopod, mesal view (A), ventral view (B), dorsal view (C), lateral view (D). Abbreviations: ca = cannula; co = coxite; pref = prefemorite; fe = femorite; sph = solenophore; sl = solenomere; su = lateral sulcus; a = process a; b = process b.

##### Diagnosis.

The species can be recognised by having sternite 5 with a rounded, highly elevated, setiferous process between coxae 4, and gonopod conformation (process a leaf–shaped, shorter than slender, spiniform process b).

##### Distribution.

Ha Giang Province (Ha Giang) (Attems 1937, 1938), Lao Cai (this study).

##### Remarks.

The species has been known only from northern Vietnam.

#### ﻿ Kronopolites montanus

Taxon classificationAnimaliaPolydesmidaParadoxosomatidae

Golovatch, 2009

F7CEF392-741C-5373-A2B2-75947A95601E

Fig. 2

Kronopolites montanus Golovatch, 2009: 121, figs 9–16; Nguyen and Sierwald 2013: 1288; Likhitrakarn et al. 2015: 31.

##### Type specimens.

ZMUM.

**Figure 2.**
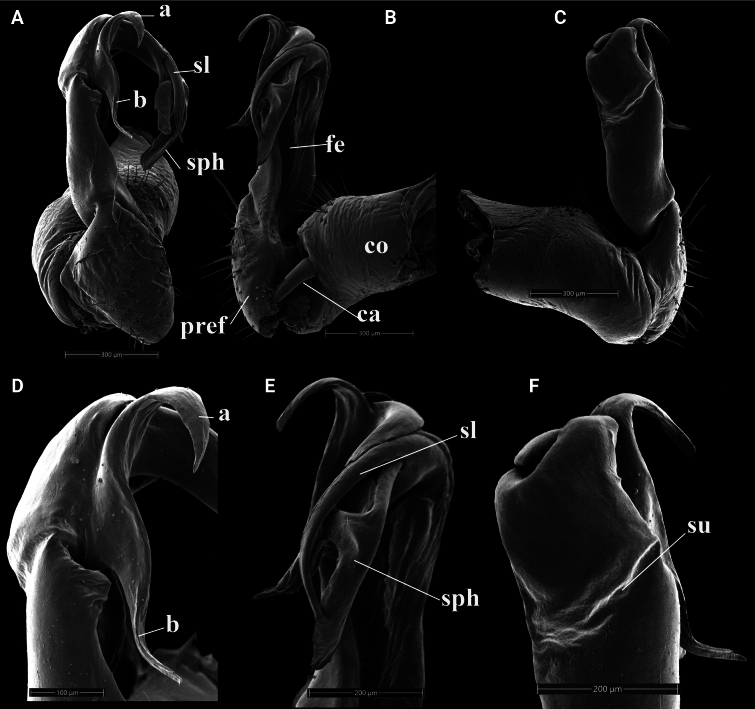
*Kronopolites montanus* Golovatch, 2009 from Ta Sua, Son La (IEBR-Myr 584). A–C. Right gonopod, ventral view (A), mesal view (B), lateral view (C); D–F. Distal part of right gonopod, ventral view (D), mesal view (E), lateral view (F). Abbreviations: ca = cannula; co = coxite; pref = prefemorite; fe = femorite; sph = solenophore; sl = solenomere; su = lateral sulcus; a = process a; b = process b.

##### Material examined.

Vietnam • **Son La Province** • 1 male; Xuan Nha Natural Reserve; 20.7211944°N, 104.67119°E; 566 m a.s.l.; residential area; 19 May 2018; Hung D. Nguyen leg.; IEBR-Myr 266 • 2 males; Ta Xua Natural Reserve; 21.33535°N, 104.49196°E; 490 m a.s.l.; bamboo forest; 9 February 2017; Son X. Le & Ha T. Vu leg.; IEBR-Myr 581 • 4 males, 3 females, 2 juveniles; same locality as IEBR-Myr 581; around Chieu village, near irrigation lake; 21.29298°N, 104.36719°E; 450 m a.s.l.; 11 February 2017; IEBR-Myr 582 • 1 male, 1 female; same locality as IEBR-Myr 581; 21.33978°N, 104.68736°E; 400 m a.s.l.; cultivated land; 10 February 2017; Son X. Le & Ha T. Vu leg.; IEBR-Myr 584 • 2 males, 1 female; same locality as IEBR-Myr 581; mixed forest; 30 May 2018; Hung D. Nguyen leg.; IEBR-Myr 752 • 2 males, 1 female; same locality as IEBR-Myr 581; mixed forest; 30 April 2018; Hung D. Nguyen leg.; IEBR-Myr 753 • 1 male; same locality as IEBR-Myr 581; natural forest; 30 May 2018; Hung D. Nguyen leg.; IEBR-Myr 757; **Lao Cai Province** • 1 male, 5 females, 2 juveniles; Van Ban District, Nam Xay commune; 22.03536°N, 104.00361°E; 600–700m a.s.l.; bamboo forest; 28 March 2005; Anh D. Nguyen leg.; IEBR-Myr 110 • 1 male; same locality as IEBR-Myr 110; bamboo forest; 900m a.s.l.; 12 April 2005; Anh D. Nguyen leg.; IEBR-Myr 112 • 1 male; Hoang Lien National Park; 22.41475°N, 103.78133°E; 2,000 m a.s.l.; nature forest;20–29 March 2007; Anh D. Nguyen leg.; IEBR-Myr 186 • 1 male; same locality as IEBR-Myr 186; 1,900m a.s.l.; bamboo forest; 20–29 March 2007; Anh D. Nguyen leg.; IEBR-Myr 189 • 1 male, 1 female; Hoang Lien National Park; 1,526m a.s.l.; mixed forest; 16 March 2018; Hung D. Nguyen leg.; IEBR-Myr 763; **Phu Tho Province** • 1 male; Xuan Son National Park, on the way to Lang village; 21.12521°N, 104.97299°E; 16 March 2006; Nguyen Van Quang leg.; IEBR-Myr 26 • 1 male, 1 female; same locality as the IEBR-Myr 26; Lap village; 21.13776°N, 104.93149°E; 17 January 2006; Nguyen Van Quang leg.; IEBR-Myr 114 • 1 male, 1 female; Xuan Son National Park, Lap village; 21.13776°N, 104.93149°E; pitfall trapping; 6 April 2011; An & Luong leg.; IEBR-Myr 173.

##### Diagnosis.

The species can be recognised by black-brown colouration, the absence of cones or lamina between coxae 4 on sternite 5, and gonopod conformation (process a shorter and coiled, while process b longer, straight, digitiform and subhelicoid). The species differs from its close congener, *K. ramosus*, in shape and length of process a and b (a short, wider coiled process a and a longer, straight, digitiform, subhelicoid process b in *K. montanus* vs a short and thin, curved, process a with an apical hook and a longer, straight, acuminate process b in *K. ramosus*).

##### Distribution.

Lao Cai Province (Sapa) (Golovatch 2009); Son La Province (Xuan Nha NR; Ta Sua NR); Phu Tho Province (Xuan Son NP) (this study).

##### Remarks.

The species is widely distributed in northwestern Vietnam.

#### ﻿ Kronopolites ramosus

Taxon classificationAnimaliaPolydesmidaParadoxosomatidae

Golovatch & Semenyuk, 2021

229EDB61-ACDB-56A9-A580-65B36AD7244F

Fig. 3

Kronopolites ramosus Golovatch & Smenyuk, 2021: 479, figs 31–46.

##### Type specimens.

ZMUM.

**Figure 3.**
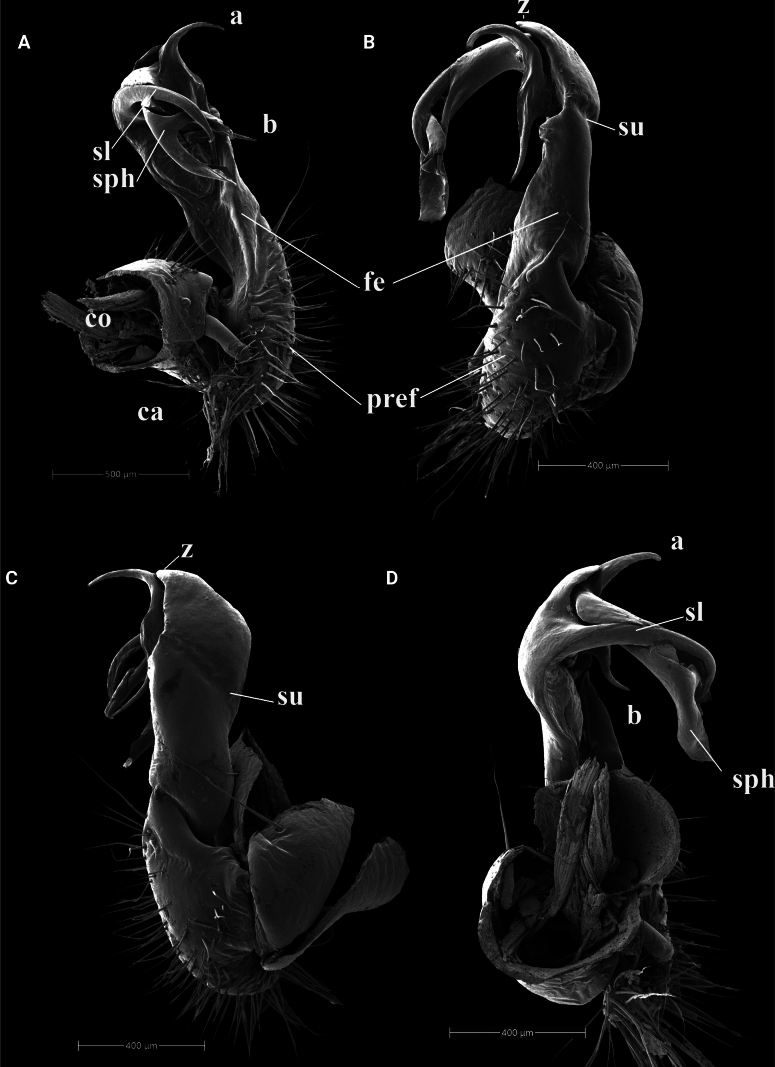
*Kronopolites ramosus* Golovatch & Semenyuk, 2021. IEBR-Myr 174, left gonopod, mesal view (A), ventral view (B), lateral view (C), dorsal view (D). Abbreviations: ca = cannula; co = coxite; pref = prefemorite; fe = femorite; sph = solenophore; sl = solenomere; su = lateral sulcus; a = process a; b = process b; z = spine z.

##### Material examined.

Vietnam • **Ha Tinh Province** • 1 male, 1 female; Huong Son District, Son Kim commune; 18.46268°N, 105.15382°E; 350 m a.s.l.; natural forests; 3 May 2004; Anh D. Nguyen leg.; IEBR-Myr 111; Nghe An Province • 3 males, 6 females, 1 juvenile; Pu Mat National Park; Khe Thoi; 19.07991°N, 104.63714°E; closed forest, near stream; 4–10 April 2011; Anh D. Nguyen leg; IEBR-Myr 175 • 7 males, 5 females; same as the sample IEBR-Myr 175; IEBR-Myr 176 • 2 females, 2 juveniles; Pu Mat National Park, Thac Chem waterfall; 18.97150°N, 104.80081°E; 430 m a.s.l.; evergreen closed forest; 4–10 April 2011; Anh D. Nguyen leg.; IEBR-Myr 199 • 5 males, 3 females; Pu Mat National Park; same as the sample IEBR-Myr 175; IEBR-Myr 553 • 1 male; Pu Mat National Park; same as the sample IEBR-Myr 175; IEBR-Myr 174.

##### Diagnosis.

The species can be distinguished by having a uniformly dark colouration and yellow legs, sternite 5 without any processes between male coxae 4, gonopod process a unciform while process b straight, both a and b slender and acuminate, sharing a broad lobe-shaped base.

##### Distribution.

Nghe An Province (Pu Mat NP) (Golovatch and Semenyuk 2021).

##### Remarks.

The species has been known only from Vietnam.

##### Remarks.

As mentioned above, two species, *K. montanus* and *K. ramosus*, show high similarity in morphology, especially gonopod conformation (see Figs 2, 3). The two species can be differentiated based on their gonopod processes: *K. montanus* has a short, wider coiled process a and a longer, straight, digitiform, subhelicoid process b, while *K. ramosus* has a short and thin, curved, process a with an apical hook and a longer, straight, acuminate process b. In addition, two species have high significant genetic divergence (COI distance = 11.6%). Therefore, two species are clearly distinguished from each other.

#### ﻿ Kronopolites biagrilectus

Taxon classificationAnimaliaPolydesmidaParadoxosomatidae

Hoffman, 1963

A00CB842-1B0F-5783-9478-9767295777C3

Fig. 4

##### Material examined.

Vietnam • 1 male; Dien Bien Province, Muong Nhe Natural Reserve; 22.29225°N, 102.38603°E; 784 m a.s.l.; natural forest; 7 May 2018; Hung D. Nguyen leg.; IEBR-Myr 756.

**Figure 4.**
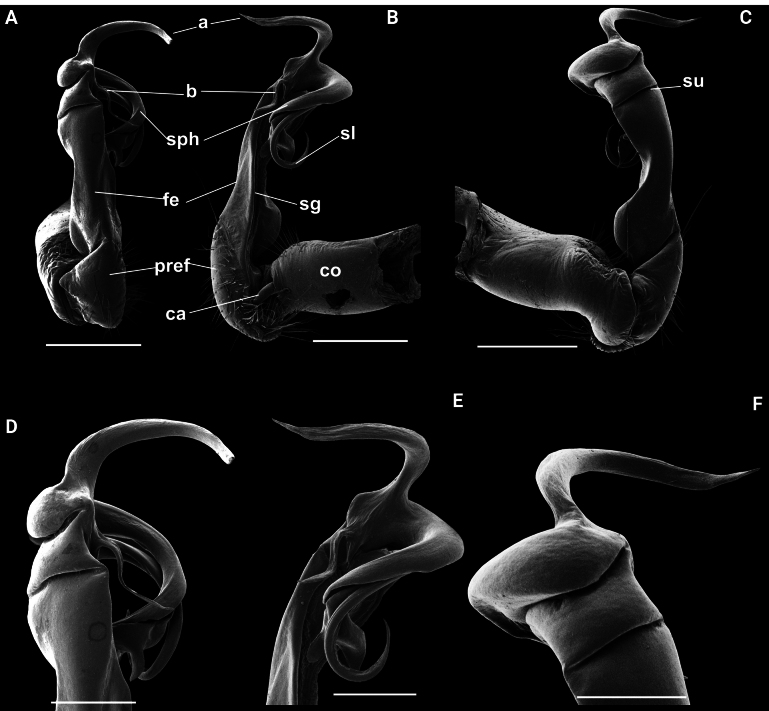
*Kronopolites biagrilectus* Hoffman, 1963 from Dien Bien (IEBR-Myr 756). A–C. Left gonopod, ventral view (A), mesal view (B), lateral view (C); D–F Distal part of left gonopod, ventral view (D), mesal view (E), lateral view (F). Abbreviations: ca = cannula; co = coxite; pref = prefemorite; fe = femorite; sph = solenophore; sl = solenomere; sg = seminal groove; su = lateral sulcus; a = process a; b = process b.

##### Diagnosis.

The species is similar to *K. acuminatus* (Attems, 1937), but distinguished by the form of gonopod processes a and b (process a shorter than process b in *K. acuminatus*, *vice versa* in *K. biagrilectus*).

##### Distribution.

Widely distributed in southern China (Golovatch 2020) and Vietnam (this study).

##### Remarks.

The species was proposed as a subspecies, *K. acuminatus biagrilectus* Hoffman, 1963, and considered as a northern form of *K. acuminatus acuminatus*. The only difference between the two species is the length ratio of processes a and b. The species is recorded herewith from Vietnam for the first time. Our specimen fits well the description of Hoffman (1963).

#### ﻿ Kronopolites contrastus sp. nov.

Taxon classificationAnimaliaPolydesmidaParadoxosomatidae

8889CB4C-39B0-5668-9152-F2909B07B2B6

https://zoobank.org/BD192848-AC47-44DB-9987-44D705BAE22B

Figs 5 , 6 , 7 , 8

##### Material examined.

***Holotype***: Vietnam • 1 male; Tuyen Quang Province, Cham Chu Nature Reserve; 22.20248°N, 105.11154°E; limestone forest; July 2018; Dai D. Nguyen leg.; IEBR-Myr 718.

##### Diagnosis.

The species differs from its congeners in colouration pattern (tergites mostly dark while other parts of body whitish yellow), gonopod conformation (lamina l present laterally, subrectangular; processes a and b both leaf-shaped, but pointed; a shorter than b in length; process a subhelicoid. Solenophore clearly curved, long, expanded distomesally, bipartite. Solenomere longer than solenophore, ribbon-shaped, coiled).

##### Description.

***Size***: Length 48.38 mm, width of midbody pro- and metazona 3.41 and 4.87 mm, respectively.

**Figure 5.**
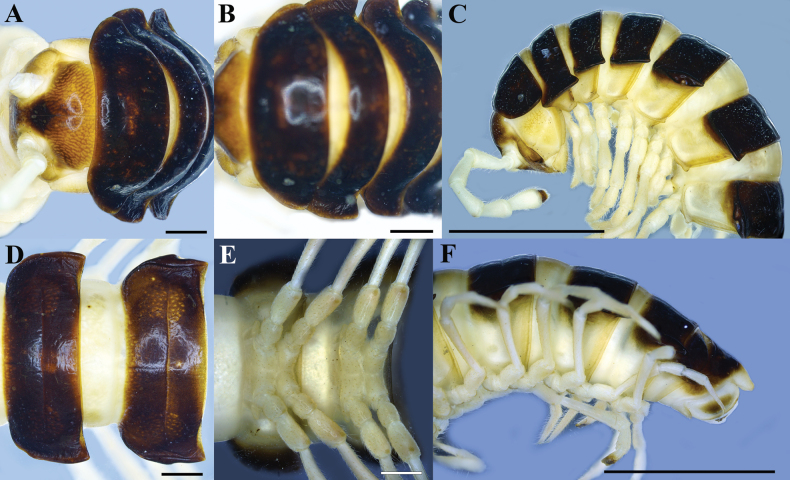
*Kronopolites contrastus* sp. nov. Holotype (IEBR-Myr 718). A. Head and collum, dorsal view; B. Collum, dorsal view; C. Anterior part of body, lateral view; D, E. Body rings 8–9, dorsal view (D), ventral view (E); F. Posterior part of body, lateral view. Scale bars: 1 mm (A, B, D, E) 5 mm (C, F).

**Figure 6.**
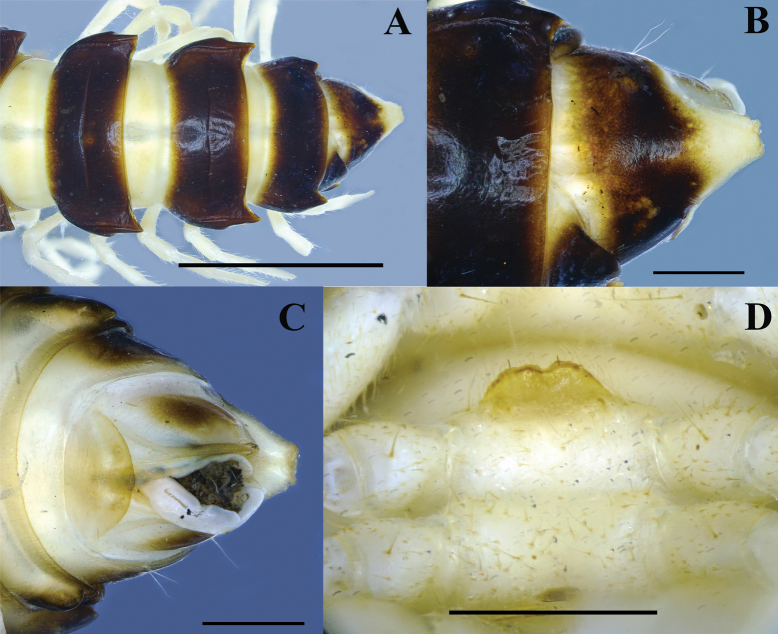
*Kronopolites contrastus* sp. nov. Holotype (IEBR-Myr 718). A. Posterior part of body, dorsal view; B, C. Telson, dorsal view (B), ventral view (C); D. Sternum 5, subventral view. Scale bars: 1 mm (B, C); 5 mm (A, D).

***Colouration***: tergites mostly dark; head blackish brown; prozonae, pleurites, sternites, antennae, telson, and legs whitish yellow (Figs 5, 6).

***Head***: Clypeolabral region and vertex densely setose, epicranial suture distinct. Antennae moderately long (Fig. 5A, B), extending behind body segment 3 when stretched laterally; antennomere 2=3=4>6>5>7=1, antennomere 7 with four conical sensories.

***Collum***: traces of setae hardly seen; lateral incisions absent; caudal corner of paraterga very broadly rounded, declined ventrad, produced behind rear tergal margin (Fig. 5A, B).

***Body rings***: In width, segment 4 < 3 < head < 5 < collum < body ring 2 < 6–17, thereafter body gently and gradually tapering. Tegument smooth and shining, prozonae finely shagreened, metaterga finely rugulose (Figs 5A–D, 6A, B); surface below paraterga finely microgranulate (Fig. 5C, F). Tergal setae all broken, traces hardly visible. Axial line distinct on anterior halves of metazonae (Figs 5D, 6A). Transverse sulcus usually distinct (Fig. 5D), slightly incomplete on body rings 4 and 19, complete on metaterga 5–18, narrow, line-shaped, shallow, reaching bases of paraterga. Stricture between pro– and metazonae evident, broad and deep, ribbed at bottom down to base of paraterga (Figs 5D, 6A). Pleurosternal carinae complete crests with a sharp caudal tooth on body rings 2–7 (Fig. 5C, F), thereafter increasingly strongly reduced until body ring 17.

***Paraterga*** strongly developed, lying rather high (at upper 1/3 of body), slightly upturned, but lying below dorsum; anterior edge broadly rounded and narrowly bordered, fused to callus; caudal corner very narrowly rounded, starting from segment 15 extending increasingly well beyond rear tergal margin (Figs 5C, D, F, 6A, B); lateral edge without incisions; posterior edge nearly straight. Calluses on paraterga narrow, delimited by a sulcus both dorsally and ventrally. Ozopores evident, lateral, lying in an ovoid groove at ~1/4 in front of posterior edge of metaterga (Figs 5C, D, 6A).

***Telson***: Epiproct (Figs 5F, 6A–C) conical, flattened dorsoventrally, with two small apical papillae; tip subtruncate; pre–apical papillae small, lying close to tip. Hypoproct sub–semicircular, setiferous knobs at caudal edge small and well–separated (Fig. 6C).

***Sterna***: densely setose, without modifications except sternum 5^th^ with a bifid tongued–shaped, setose cone between male coxae 4 (Fig. 6D).

***Legs*** (Fig. 6C, F): rather long and slender, midbody ones ~1.2–1.3 times as long as body height; prefemora without modifications, tarsal brushes present on pregonopodal legs.

**Figure 7.**
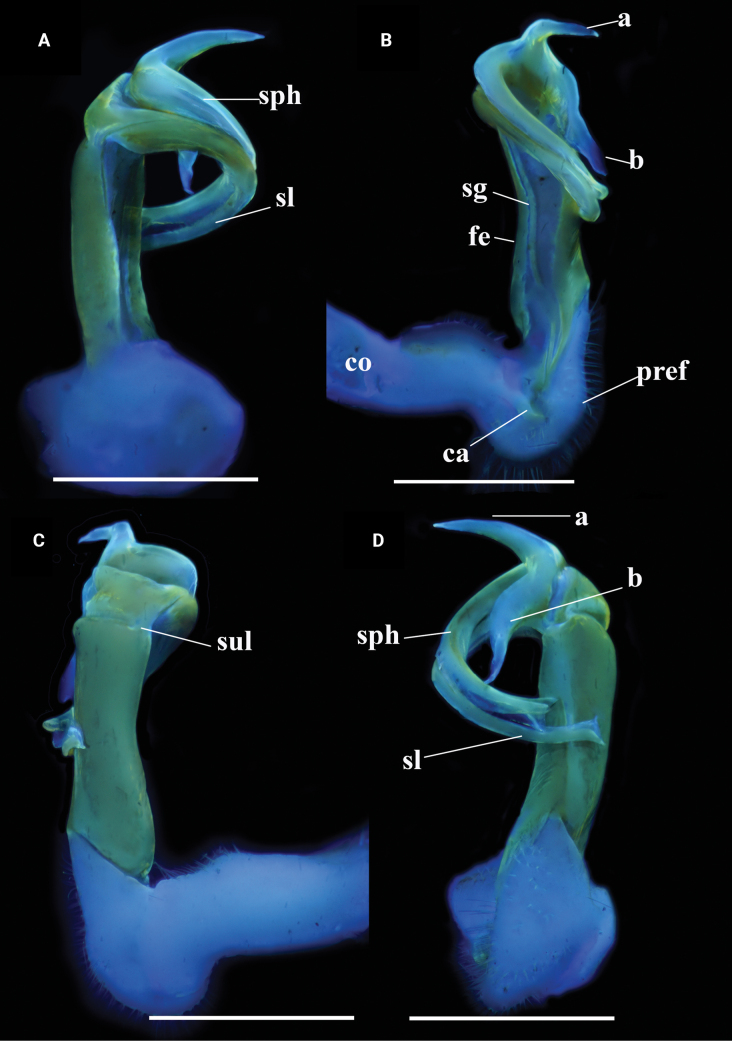
*Kronopolites contrastus* sp. nov. Holotype (IEBR-Myr 718). UV images, right gonopod, dorsal view (A), mesal view (B), lateral view (C), ventral view (D). Scale bars: 1 mm. Abbreviations: ca = cannula; co = coxite; pref = prefemorite; fe = femorite; sph = solenophore; sl = solenomere; sg = seminal groove; su = lateral sulcus; a = process a; b = process b.

**Figure 8.**
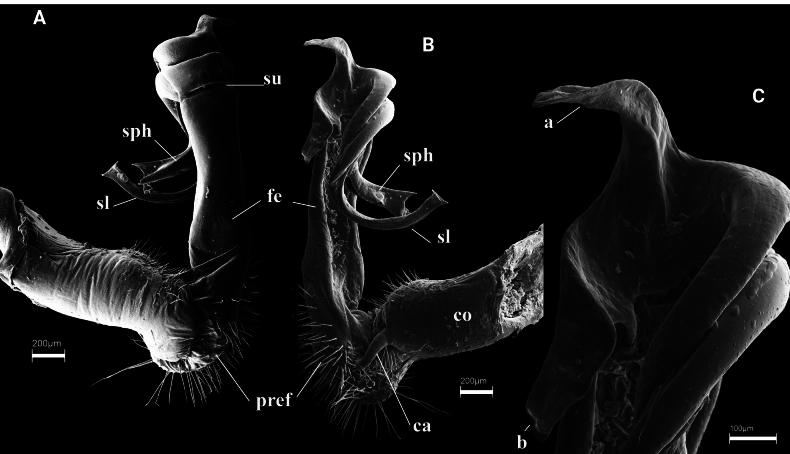
*Kronopolites contrastus* sp. nov. Holotype (IEBR-Myr 718), left gonopod, lateral view (A), mesal view (B); distal part of left gonopod, mesal view (C).

***Gonopods*** (Figs 7, 8) typical *Kronopolites* species; coxite (co) long, subcylindrical, a little curved caudad, sparsely setose distoventrally. Prefemur (pref) densely setose, ~1/3 as long as femorite + postfemoral part. Femorite (fe) long, cylindrical, slightly constricted medially, simple without any modifications; mesal side strongly grooved; lateral side with a distinct transverse sulcus demarcating the postfemoral region. Postfemoral part well developed; lamina l present laterally, subrectangular; processes a and b both leaf-shaped, but pointed; a shorter than b in length; process a subhelicoid. Solenophore clearly curved, long, expanded distomesally, bipartite. Solenomere longer than solenophore, ribbon-shaped, coiled.

##### Etymology.

*Contrastus*, an adjective epithet is used to emphasise the contrast colouration of body.

##### Remarks.

The new species is similar to *K. fuscocingulatus* from northern Thailand by having a sternal lobe between male coxae 4, and processes a and b of gonopod being nearly independent, subequal in length. However, two species are distinguished by shape of sternal lobe (bifid tongue-shaped vs roundly subquadrate), and shape of process a and b (spiniform, blunt, short, and stout vs ribbon-shaped, blunt, slender and long).

#### ﻿ Kronopolites serratus sp. nov.

Taxon classificationAnimaliaPolydesmidaParadoxosomatidae

02E7C2BC-C3ED-5697-A8FC-4ED8B07F8982

https://zoobank.org/7093E093-191E-4B1E-99A7-84003A5BA0C0

Figs 9 , 10 , 11

##### Material examined.

***Holotype***: Vietnam • 1 male; Lao Cai Province, Hoang Lien National Park, Thac Bac waterfall; 22.36350°E, 103.77754°E; 1,950 m a.s.l.; regenerated forest; 28 November 2005; Anh D. Nguyen leg.; IEBR-Myr 109.

##### Diagnosis.

The new species is similar to *K. montanus* Golovatch, 2009, but differs in the following characters: gonopod process a curved, serrated, acuminate leaf-shaped, as long as distally serrated, ribbon-shaped process b.

##### Description.

***Size***: length 32.01 mm, width of midbody pro- and metazona 2.67 mm and 3.67 mm, respectively.

**Figure 9.**
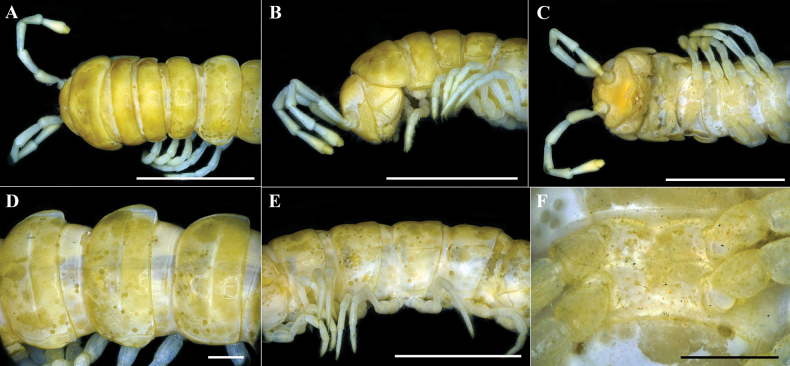
*Kronopolites serratus* sp. nov. Holotype (IEBR-Myr 109). A–C. Anterior part of body, dorsal view (A), lateral view (B), ventral view (C); D, E. Body rings 9–11, dorsal view (D), lateral view (E); F. Sternum 5, ventral view. Scale bars: 5 mm (A–C, E); 1 mm (D, F).

***Colouration*** (Fig. 9): almost uniformly brownish yellow after long preservation in ethanol.

***Head*** (Fig. 9A–C): Clypeolabral region and vertex densely setose, epicranial suture distinct. Antennae claviform, moderately long (Fig. 9A–C), extending behind body segment 3 when stretched laterally; antennomere 2=3=4>5>6>7=1, antennomere 7 with four conical sensories.

***Collum***: traces of setae hardly seen; lateral incisions absent; caudal corner of paraterga very broadly rounded, declined ventrad, produced behind rear tergal margin (Fig. 9A, B).

***Body rings***: In width, segment 4 < 3 < head < 5 < segment 2 < collum < 6–17, thereafter body gently and gradually tapering. Tegument (Fig. 9A–E) smooth and shining, prozonae finely shagreened; surface below paraterga finely rugulose. Tergal setae all broken, traces hardly seen. Axial line visible, especially on midbody tergites. Transverse sulcus usually distinct (Fig. 9A), slightly incomplete on body rings 4 and 19, complete on metaterga 5–18, narrow, line–shaped, shallow, reaching bases of paraterga. Stricture between pro– and metazonae evident, broad and deep, striolated at bottom down to base of paraterga (Fig. 9D). Pleurosternal carinae complete crests on body rings 2–7; thereafter, increasingly strongly reduced until body ring 17.

***Paraterga*** strongly developed (Fig. 9A, D), lying below dorsum; anterior edge broadly rounded and narrowly bordered, fused to callus; caudal corner very narrowly rounded; lateral edge without incisions; posterior edge nearly straight. Calluses on paraterga narrow, delimited by a sulcus both dorsally and ventrally. Ozopores (Fig. 9D, E) evident, lateral, lying in an ovoid groove at ~1/4 in front of posterior edge of metaterga.

***Telson***: damaged.

***Sterna***: densely setose, without modifications (Fig. 9C, F).

***Legs***: rather long and slender, midbody ones ~1.2–1.3 times as long as body height; prefemora without modifications, tarsal brushes present only on pregonopodal legs

**Figure 10.**
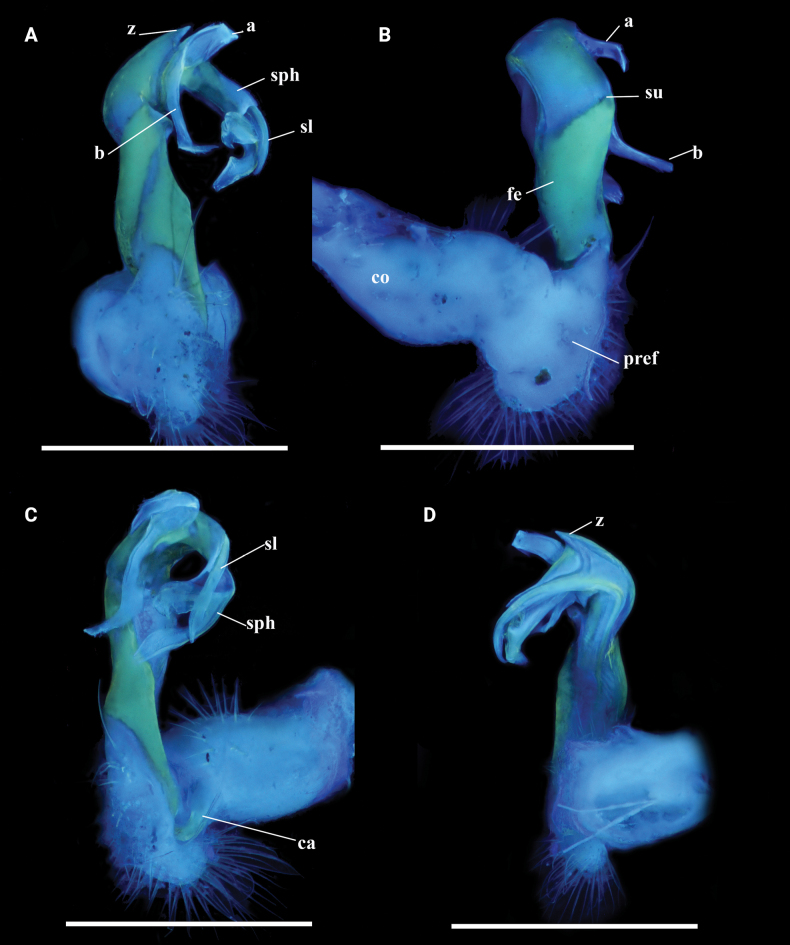
*Kronopolites serratus* sp. nov. Holotype (IEBR-Myr 109). UV images, left gonopod, ventral view (A), lateral view (B), mesal view (C), dorsal view (D). Abbreviations: ca = cannula; co = coxite; pref = prefemorite; fe = femorite; sph = solenophore; sl = solenomere; sg = seminal groove; su = lateral sulcus; a = process a; b = process b; z = spine z. Scale bars: 1 mm.

**Figure 11.**
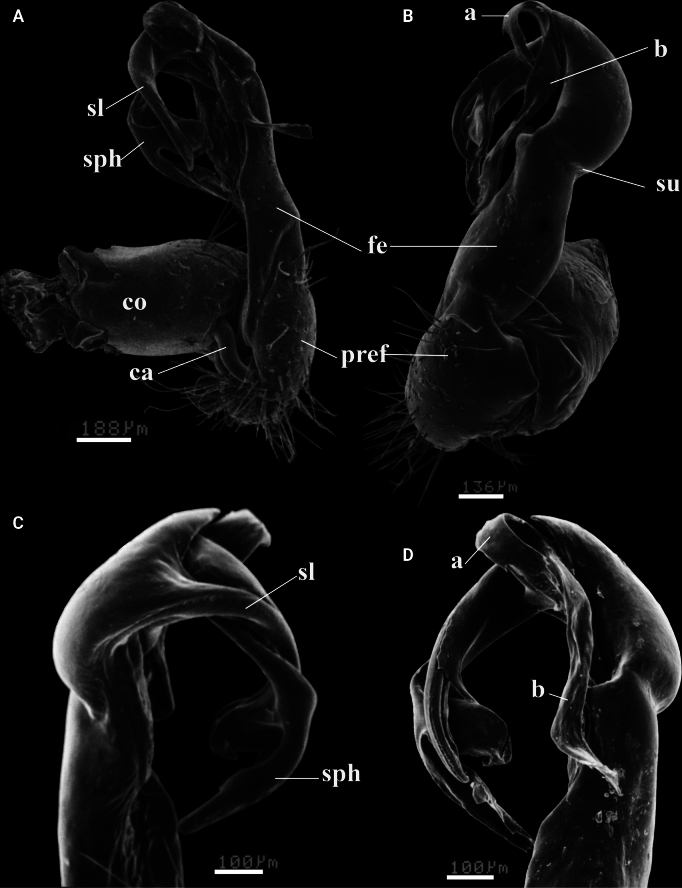
*Kronopolites serratus* sp. nov. Holotype (IEBR-Myr 109). A, B. Right gonopod, mesal view (A), ventral view (B); C, D. Distal part of left gonopod, dorsal view (C), mesoventral view (D). Abbreviations: ca = cannula; co = coxite; pref = prefemorite; fe = femorite; sph = solenophore; sl = solenomere; su = lateral sulcus; a = process a; b = process b.

***Gonopods*** (Figs 10, 11) a typical *Kronopolites* species; coxite (co) stout, subcylindrical, sparsely setose distoventrally. Prefemur (pref) densely setose, ~1/3 as long as femorite + postfemoral part. Femorite somewhat stout, evidently grooved mesally, demarcated from postfemoral region by a distinct oblique sulcus laterally. Lamina l present, producing into a small spine z distally; process a leaf-shaped, mesal margin serrated, clearly shorter than distally-serrated, ribbon-shaped process b; solenophore well developed, bipartite, longer than a short flagelliform solenomere which parly sheathed by solenophore.

##### Remarks.

Although the new species is described based on only a male, its gonopod distinctly differs from that of *K. montanus* in gonopod process a being curved, serrated, acuminate leaf-shaped, as long as distally serrated, ribbon-shaped process b. Both species are also found in Hoang Lien National Park at the high elevation of more than 1,900 m a.s.l.

##### Etymology.

*Serratus*, an adjective epithet, is used to emphasise the serrated process a and b of the gonopod.

### ﻿Molecular analysis

The dataset includes a 590 bp fragment of the COI from 28 samples that represent 14 different sulciferinine morphospecies, along with a root species, *Antheromorpha festiva* (Table 2). The mean K2P distance among sulciferinine morphospecies was 17.0%. The genetic difference between the root and other Vietnamese sulciferinine morphospecies ranged from 19.1% to 23.4% (Table 3).

**Table 2.** Voucher species and accession numbers deposited in GenBank.

No.	Species	Locality	Voucher	Accession numbers (COI)	Source
1	*Kronopolites montanus* Golovatch, 2009	Xuan Nha NR, Son La Prov.	IEBR-Myr 266	PV892454	This study
2	*Kronopolites montanus* Golovatch, 2009	Ta Xua NR, Son La Prov.	IEBR-Myr 582	PV892456	
3	*Kronopolites ramosus* Golovatch & Semenyuk, 2021	Pu Mat NP, Nghe An Prov.	IEBR-Myr 174	PV892452	
4	*Kronopolites ramosus* Golovatch & Semenyuk, 2021	Pu Mat NP, Nghe An Prov.	IEBR-Myr 175	PV892453	
5	*Kronopolites ramosus* Golovatch & Semenyuk, 2021	Pu Mat NP, Nghe An Prov.	IEBR-Myr 553	PV892455	
6	*Kronopolites biagrilectus* Hoffman, 1963	Muong Nhe NR, Dien Bien Prov.	IEBR-Myr 756	PV892457	
7	*Sellanucheza grandis* (Golovatch, 1984)	Pu Mat National Park, Nghe An Prov.	IEBR-Myr 177	KR818296	GenBank
8	*Sellanucheza grandis* (Golovatch, 1984)	Huong Son District., Ha Tinh Prov.	IEBR-Myr 59	KR818293	
9	*Sellanucheza hoffmani* (Nguyen, 2011)	Phong Nha – Ke Bang National Park, Quang Binh Prov.	IEBR-Myr 182	KR818298	
10	*Sellanucheza variata* (Attems, 1953)	Duc Xuan commune, Bac Quang Distr., Ha Giang Prov.	IEBR-Myr 515	OM919709	
11	*Oxidus gigas* (Attems, 1953)	Sapa, Lao Cai Prov.	IEBR-Myr 133	MG669364	
12	*Oxidus gigas* (Attems, 1953)	Duc Xuan commune, Bac Quang Distr., Ha Giang Prov.	IEBR-Myr 516	KX096928	
13	*Oxidus gracilis* (CL Koch, 1847)	Okinawa Isls., Japan	IEBR-Myr H466	KX096924	
14	*Oxidus gracilis* (CL Koch, 1847)	Okinawa Isls., Japan	IEBR-Myr H471	KX096925	
15	*Oxidus gracilis* (CL Koch, 1847)	U.S.A.	IEBR-Myr USA	KX096931	
16	*Oxidus riukiuria* (Verhoeff, 1940)	Okinawa Isls., Japan	IEBR-Myr H500	KX096926	
17	*Oxidus riukiuria* (Verhoeff, 1940)	Okinawa Isls., Japan	IEBR-Myr H500J	KX096927	
18	*Tylopus crassipes* Golovatch, 1984	Nam Xay commune, Van Ban District, Lao Cai Prov.	IEBR-Myr 92	KX096920	
19	*Tylopus hilaroides* Golovatch, 1984	Tam Dao NP, Vinh Phuc Prov.	IEBR-Myr SVE55	MW384903	
20	*Tylopus hilaroides* Golovatch, 1984	Cuc Phuong NP, Ninh Binh Prov.	IEBR-Myr SVE173	MW384904	
21	*Tylopus hilaroides* Golovatch, 1984	Ba Vi NP, Hanoi	IEBR-Myr SVE149	MW384905	
22	*Tylopus hilaroides* Golovatch, 1984	Cuc Phuong NP, Ninh Binh Prov.	IEBR-Myr 543	MW384914	
23	*Tylopus hilaroides* Golovatch, 1984	Cuc Phuong NP, Ninh Binh Prov.	IEBR-Myr 198	MW384918	
24	*Tylopus roseiparaterga* Nguyen, 2012	Tam Dao NP, Vinh Phuc Prov.	IEBR-Myr 185A	KX096923	
25	*Tylopus roseiparaterga* Nguyen, 2012	Ba Vi NP, Hanoi	IEBR-Myr SVE70	MW384902	
26	*Tylopus sapaensis* Nguyen, 2012	Hoang Lien NP, Lao Cai Prov.	IEBR-Myr 93	MW384908	
27	*Tylopus spinisterna* Nguyen, 2012	Bi Doup – Nui Ba NP, Lam Dong Prov.	IEBR-Myr 234	MW384916	
28	*Antheromorpha festiva* (Brölemann, 1916)	Yon Don NP, Dak Lak Prov.	IEBR-Myr 519	MG669361	

**Table 3.** Pairwise nucleotide difference (Kimura 2-parameter model) over sequence pairs between sulciferinine species. Numbers in bold are intraspecific divergences.

No	Species	1	2	3	4	5	6	7	8	9	10	11	12	13	14
**1**	*Antheromorpha festiva*														
**2**	*Kronopolites biagrilectus*	22.0%													
**3**	*Kronopolites montanus*	18.5%	17.7%	**9**%											
**4**	*Kronopolites ramosus*	21.6%	16.6%	11.6%	**2**%										
**5**	*Oxidus gigas*	21.8%	22.1%	22.4%	23.4%										
**6**	*Oxidus gracilis*	21.8%	22.9%	18.9%	18.1%	14.0%									
**7**	*Oxidus riukiarius*	20.9%	19.5%	17.6%	17.4%	15.1%	12.5%								
**8**	*Sellanucheza grandis*	20.2%	18.1%	16.3%	16.6%	20.3%	16.9%	15.5%							
**9**	*Sellanucheza hofmani*	20.9%	19.5%	18.2%	18.5%	22.3%	18.6%	17.6%	12.4%						
**10**	*Sellanucheza variata*	19.1%	18.5%	17.2%	18.5%	18.7%	18.0%	16.1%	11.7%	12.4%					
**11**	*Tylopus crassipes*	22.2%	20.4%	22.1%	20.5%	18.5%	20.2%	19.7%	20.1%	19.9%	19.8%				
**12**	*Tylopus hilaroides*	23.2%	21.4%	19.9%	19.4%	19.5%	16.7%	14.4%	17.0%	17.5%	16.1%	17.8%			
**13**	*Tylopus roseiparaterga*	22.4%	18.1%	19.8%	16.6%	21.5%	18.1%	17.8%	17.2%	18.0%	16.5%	13.4%	17.8%		
**14**	*Tylopus sapaensis*	21.1%	21.5%	20.4%	19.3%	16.7%	15.8%	15.1%	17.2%	15.4%	14.5%	18.2%	14.6%	16.9%	
**15**	*Tylopus spinisterna*	19.7%	18.5%	18.7%	18.4%	20.4%	19.2%	15.0%	15.7%	16.5%	14.3%	16.9%	15.8%	16.9%	17.1%

The intraspecific distance of the *Kronopolites* species varied from 2%. (*K. ramosus*) to 9% (*K. montanus*) The intraspecific K2P distance was clearly lower than the average distance between sulciferine morphospecies (17.0%). The interspecific distance between *Kronopolites* species ranged from 11.6% to 17.7%. Unfortunately, only samples of three *Kronopolites* species could be sequenced for the COI fragment.

**Figure 12.**
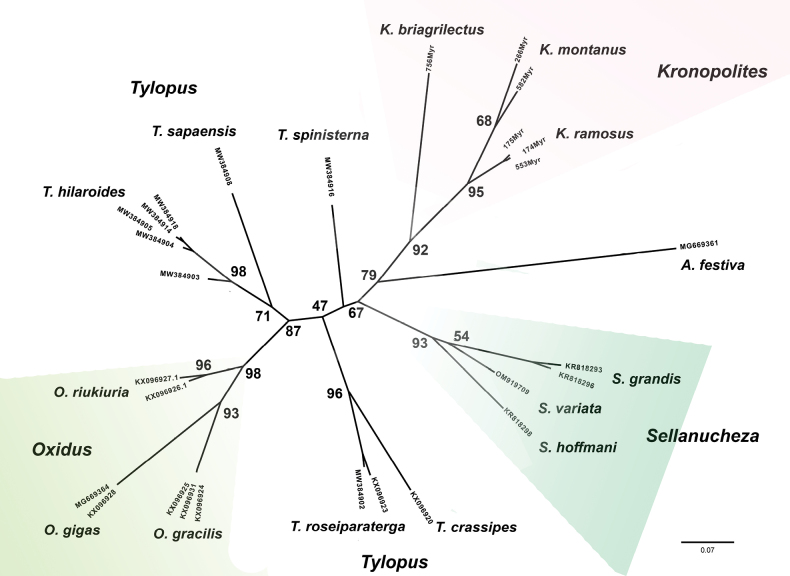
Phylogenetic diagram of Vietnamese sulciferinine species inferred from a 590 bp fragment of COI using maximum likelihood analysis. Numbers at nodes are bootstrap values.

The analysis resulted in each species formed a monophyletic clade with high bootstrap support values. Three *Kronopolites* are separated in different clades supported by high bootstrap values (92%–95%). Almost all examined sulciferinine genera were monotypic and formed their own clades except the genus *Tylopus* (Fig. 12).

### ﻿An identification key to species of the genus *Kronopolites* Attems, 1914

Updated from Golovatch (2020), based on gonopod morphology and other male structures.

**Table:** 

1	Colouration with a strongly contrasting pattern, some parts of body segments being much paler, some other ones (regardless of largely yellowish venter and legs) much darker	**2**
–	Colouration more uniform, usually brown to brown-blackish, regardless of largely yellowish venter and legs; more rarely generally pale	**7**
2	Paraterga relatively poorly developed, set low (mostly at ca. upper 1/3 of segments), caudal corners of midbody paraterga not projecting past tergal margin, at most narrowly rounded	**3**
–	Paraterga relatively well developed, mostly set higher, caudal corners of midbody paraterga clearly produced past caudal tergal margin, acuminate	**5**
3	Sternal lobe between male coxae 4 roundly subquadrate or bifid tongue–shaped; male sternal cones absent; processes a and b of gonopod nearly independent, subequal in length	**4**
–	Sternal lobe between male coxae 4 concave, with a paramedian pair of small knobs between rounded apicolateral corners; male sternal cones present; processes a and b of gonopod on a broad common stem, b sharp, axe-shaped and considerably shorter than a. China	***K. svenhedini***
4	Sternal lobe between male coxae 4 roundly subquadrate; processes a and b of gonopod nearly independent, both ribbon-shaped, blunt, slender and long. Northern Thailand	***K. fuscocingulatus***
–	Sternal lobe between male coxae 4 bifid tongue-shaped; processes a and b of gonopod spiniform, blunt, short, and stout. Northern Vietnam	***K. contrastus* sp. nov.**
5	Colouration dark brown with yellow paraterga; paraterga largely wing-shaped and upturned; paramedian sternal cones between male coxae 3–5, the largest between 4^th^; processes a and b of gonopod short and small, sharing a very distinct common stem. Kashmir Himalaya	***K. occidentalis***
–	Colour pattern different, rear halves of prozonae and fore halves of metazonae usually black–brown, remaining parts yellowish; paraterga neither wing-shaped nor upturned, lying well below dorsum; a single sternal lobe present only between male coxae 4; processes a and b of gonopod longer and slenderer, their shared base far less conspicuous	**6**
6	Process a of gonopod somewhat shorter than process b. Northern Vietnam	***K. acuminatus***
–	Process a of gonopod somewhat longer than process b. Southern China	***K. biagrilectus***
7	General colouration pale, paraterga yellow, metaterga pale brown; paraterga very strongly developed, wing-shaped, mostly upturned; metaterga clearly coriaceous; processes a and b of gonopod subequal, both forming a short, strong, sharp fork on a distinct stem. Nepal	***K. coriaceus***
–	General colouration dark; paraterga neither wing-shaped nor upturned; metaterga never coriaceous, at most rugulose/vermiculate; processes a and b of gonopod clearly different in shape and/or length	**8**
8	A pair of small paramedian cones between male coxae 4; process a of gonopod almost twice as long as process b. Northern Laos	***K. lunatus***
–	Either a single lobe between male coxae 4 or a lobe absent; process a of gonopod either subequal in length to or almost half as long as process b	**9**
9	Processes a and b of gonopod subequal in length. Northern Taiwan	***K. formosanus***
–	Process a of gonopod almost half as long as process b	**10**
10	Neither a lobe between male coxae 4 nor male tarsal brushes. Northern Vietnam	**11**
–	A single lobe between male coxae 4; male tarsal brushes present. Southwestern China	**13**
11	Both process a and b spiniform, acuminate. Both a and b sharing a very broad lobe-shaped base	**12**
–	Process a leaf–shaped, serrated marginally while process b long, ribbon-shaped, distally serrated. Both processes a and b sharing a small base	***K. serratus* sp. nov.**
12	Process a short and coiled while process b longer straight, digitiform and subhelicoid. Northern Vietnam	***K. montanus***
–	Process a curved, short, apical hook while process b longer straight, acuminate. Northcentral Vietnam	***K. ramosus***
13	Process a of gonopod a small, pointed, leaf-shaped lobe, while b a slender and acuminate ribbon	***K. davidiani***
–	Process a of gonopod a curved, rather short, apically pointed ribbon nearly as long as a subtriangular b bearing a sharp basal spine with a mesal rib near base	***K. typicus***

## ﻿Discussion

With the description of two new species, the number of *Kronopolites* species increases to 16 species, distributed from northern India in the west to Taiwan in the east, and from southern China in the north to northcentral Vietnam in the south. No species have been found in southern Vietnam, central and southern Thailand, or southern Laos. Most *Kronopolites* species were found at high elevations and in dense forests in high mountains, for example *Kronopolites coriaceus* at 2,000 m a.s.l. in Nepal (Golovatch 2015) and *Kronopolites typicus* at 2,780 m on Mt. Laoheshang in Yunnan Province, China (Golovatch 2020). In the south, only two species were found at similar altitudes, namely *K. lunatus* from Xieng Khoang Province (Laos) and *K. ramosus* from Pu Mat NP (Vietnam); both species were also found at lower elevations (~ 500 m a.s.l.) (Likhitrakarn et al. 2015; Golovatch and Semenyuk 2021). Based on its distribution, *Kronopolites* is more likely to be a temperate genus. The apparent montane restriction of most *Kronopolites* species, including those in northern Vietnam, suggests that historical montane corridors may have shaped their current distribution patterns.

Among all six Vietnamese *Kronopolites* species, *K. montanus* and *K. ramosus* are highly similar in morphology, especially gonopod conformation (see Figs 2, 3). The two species can be differentiated based on their gonopod processes: *K. montanus* has a short, wider coiled process a and a longer, straight, digitiform, subhelicoid process b, while *K. ramosus* has a short and thin, curved, process a with an apical hook and a longer, straight, acuminate process b. Additionally, the separation of the two species is supported by a high genetic distance of COI (11.6%). *K. montanus* appears to be widely distributed in northern Vietnam, but the intraspecific genetic distance of COI is very high (0–9%). This suggests that *K. montanus* may be a cryptic species complex, but more surveys and genetic sequencing of additional individuals are needed to investigate this hypothesis.

## Supplementary Material

XML Treatment for Kronopolites

XML Treatment for Kronopolites acuminatus

XML Treatment for Kronopolites montanus

XML Treatment for Kronopolites ramosus

XML Treatment for Kronopolites biagrilectus

XML Treatment for Kronopolites contrastus

XML Treatment for Kronopolites serratus

## ﻿Additional information

### Use of AI

No use of AI was reported.

### Author ORCIDs

Anh D. Nguyen https://orcid.org/0000-0001-9273-0040

Tam T. T. Vu https://orcid.org/0000-0003-1145-975X

Hong Luong T. Phung https://orcid.org/0009-0001-4555-9575

Duc-Luong Tran https://orcid.org/0009-0003-5057-4619

Hung-Anh Le https://orcid.org/0009-0004-2756-2393

### Data availability

All of the data that support the findings of this study are available in the main text.
